# Interference effect of oral administration of mulberry branch bark powder on the incidence of type II diabetes in mice induced by streptozotocin

**DOI:** 10.3402/fnr.v60.31606

**Published:** 2016-06-01

**Authors:** Hua-Yu Liu, Jiang Wang, Jing Ma, Yu-Qing Zhang

**Affiliations:** 1 Silk Biotechnology Laboratory, School of Biology and Basic Medical Sciences, Soochow University, Suzhou, China; 2 National Engineering Laboratory for Modern Silk, Soochow University, Suzhou, China; 3 Henan Province Sericulture Scientific Research Institute, Henan, China

**Keywords:** mulberry branch bark, diabetes prevention, hypoglycemic, PI3K/Akt

## Abstract

**Background:**

Diabetes is a group of metabolic diseases that has become a global health problem worldwide. Many researchers have found that mulberry branches have a hypoglycemic effect, but there have been few studies or investigations regarding the use of mulberry branches to prevent the incidence of diabetes.

**Objective:**

This study aimed to investigate the potential preventive effect of mulberry branch bark powder (MBBP) from *Morus multicaulis* L against type II diabetes in mice induced by streptozotocin (STZ).

**Design:**

The normal mice were fed a diet containing 2.5, 5.0, and 10.0%, MBBP, respectively, for 2 weeks. After that, STZ (100 mg/kg) was injected into the caudal vein of these mice. These mice continued to be fed the same diet, and the fasting blood glucose (FBG) levels were monitored on the 17th and 21st days.

**Results:**

Oral administration of MBBP could effectively inhibit weight loss and maintain the FBG level. The incidence of diabetes in mice was almost inhibited by treatment with 10% MBBP. MBBP could also maintain the original antioxidant capacity and regulate the lipid metabolism in mice. An immunohistochemical assay showed that MBBP could prevent the injury of the insulin-secreting islet beta cells induced by STZ. RT-PCR also confirmed that the mRNA expression of the genes PI3K, Pdk1, Akt, and FoxO1, which were involved in the PI3K/Akt signaling pathway, hardly suffered from STZ in the 10% MBBP-dose group.

**Conclusions:**

Our results indicate that powdered mulberry branch bark has a powerful anti-diabetic effect. These results clearly illustrated that MBBP has a potential use as a health food additive in the prevention of diabetes.

Diabetes is a group of metabolic diseases that occur either when the pancreas does not produce enough insulin or when the body cannot effectively use the insulin it produces due to decreased insulin sensitivity. Structural defects in insulin can lead to chronic hyperglycemia and metabolic disorders of carbohydrates, proteins, and fat. Hence, the development of diabetes prevention is an important goal. Traditional prevention strategies are based on limiting the intake of high-calorie food and increasing exercise to lower the incidence risk of diabetes, but there have been few studies or investigations regarding the use of oral medicines or intake of health foods to prevent the incidence of diabetes (1, 2). Therefore, exploring a variety of prevention strategies to lower the risk of diabetes should be given more attention.

Mulberries (*Morus* L.) are widely cultivated in several sericulture countries, such as China, Korea, India, and Brazil. The mulberry branch is a 1- or 2-year-old branch *Ramulus mori* of mulberries (*Morus multicaulis* L) cultivated for sericulture, that is, silk farming. Other parts of the mulberry plant, such as the root, leaf, and fruit have been used as traditional Chinese medicine for a long time. In China, more than 10 million tons of mulberry branches are harvested every year (3). Most of these enormous biological resources are wasted as firewood or agricultural trash. Only a small portion is used as composite wood materials or a culture matrix for edible fungi. Therefore, new products and uses for mulberry branches should be better cultivated.

For decades, the bioactivity and pharmacological effects of the mulberry branch have been widely investigated, and it was found that the branches had various biological functions, such as hypoglycemic (4–6), hypolipidemic (7), anti-inflammatory (8, 9), antioxidant (10), and anti-tumor (11, 12) properties. In our laboratory, the extraction of flavonoids, polysaccharides, morusin, mulberroside A, and other components from mulberry branch bark have been investigated, and the results showed that these components, extracts, or isomers have bioactivity properties, such as being hypoglycemic (6, 13), hypolipidemic, anticancerous (12), and antioxidant (14). Based on these results, our experiments also tell us that the inhibitory activities of active extracts or components from mulberry branch bark against α-glucosidase *in vitro* were often an inverse proportion to its purified level. In other words, the more pure the mixture, the lower its bioactivity to inhibit α-glucosidase (polysaccharide: IC_50_ = 0.298 mg/mL (15), the obtained n-BuOH fraction: IC_50_ = 26 µg/mL (16), the 60% ethanol extract: IC_50_ =8 µg/mL (6)). Based on the extraction of active components in mulberry branch bark with organic solvents, such as alcohol, chloroform, ethyl acetate, n-propanol and others, these experiments showed that these extracts’ biological activities exhibited a serious loss *in vivo* or *in vitro*. Thus, we have attempted to use powdered mulberry branch bark for the prevention and treatment of diabetes.

The β-cell-specific toxin streptozotocin (STZ) (17) was used to induce type I or type II diabetes (T2D) in mice mainly depending on its dose. In general, the multiple, low-dose STZ approach is widely used to produce an animal model of insulin-dependent diabetes or type I diabetes involving pancreatic β-cell damage (18) and the single, higher-dose STZ injection (~100 mg/kg) is often used to induce an animal model of T2D involving more of insulin resistance (19). It is well known that oral administration of a high-fat high-sugar diet can greatly increase the incidence of STZ-induced T2D mice. Not long ago, our double experiments using the single dose of STZ were successful in producing T2D mice (6, 13). However, there are almost no reports regarding a decrease in the incidence of STZ-induced T2D mice or the prevention of STZ-induced damage to the pancreas *in vivo* by the oral administration of medical plants or their extracts. The present study aimed to investigate if the administration of mulberry branch bark powder (MBBP) protects against the incidence of T2D mice induced by STZ.

## Materials and methods

### Animals

We used male ICR mice (4 weeks old) of clean grade with body weights ranging from 18 to 22 g. The mice were obtained from the Experimental Animal Centre of Soochow University, and they were maintained under controlled conditions (50–80% humidity, 18–25°C, and a 12-h light/dark cycle). Standard chow and water were given ad libitum. The mice were allowed to acclimatize to the laboratory environment for 3 days. Mice were fed with different diets and divided into the following groups (*n*=10 per group): A: standard diet (normal control); B: standard diet (model control); C, D, and E: diets with addition of 2.5, 5.0, and 10.0% MBBP, respectively. All of the mice were fed assigned diets and water ad libitum and were weighed every 4 days. After 2 weeks, on the 13th day, STZ (100 mg/kg, from Sigma-Aldrich Fine Chemicals, Saint Louis, MO, USA) was injected into the mice through the caudal vein (20). After injection, all mice continued to be fed as above, and fasting blood glucose (FBG) levels were monitored by a glucose meter (ONETOUCH^®^UltraEasy, from Johnson & Johnson Medical (Shanghai) Ltd., China) on day 3 and day 7 after STZ injection. Then, these animals were sacrificed by decapitation. Blood samples, pancreas, and liver tissue were collected, and serum was prepared and stored at −80°C for further investigations. The serum insulin levels were measured by Insulin Assay Kit (Nanjing Jiancheng Bioengineering Institute, Nanjing, China). All animal experimental protocols used in this study were approved by the Animal Ethics Committee at Soochow University. The methods were carried out in accordance with the approved guidelines.

### MBBP preparation

The branches of the mulberry cultivar HuSang 32 (*M. multicaulis* L.) were collected from the Mulberry Garden of Soochow University, Suzhou, China, in November 2013. The bark was peeled from mulberry branches, dried at 100°C for 2 h, then pulverized twice and passed through a 200-mesh sieve to obtain the MBBP.

### Blood lipid determination

The triglyceride (TG), HDL cholesterol (HDL-C), total cholesterol (CHOL), and LDL cholesterol (LDL-C) in serum were assessed using a BS-800 chemistry analyzer (Mindray Medical International Co., Ltd., Shenzhen, China).

### Assays of the enzyme activities

In the present study, the superoxide dismutase (SOD), glutathione peroxidase (GSH-PX), and malondialdehyde (MDA) activity in liver homogenates of mice were evaluated. The SOD, GSH-PX, and MDA in this study were also performed using the total superoxide dismutase (T-SOD) assay kit (A001-1), GSH-PX assay kit (A005), and MDA assay kit (A003-1), according to the manufacturer's protocols. The absorbance of samples was determined at 550 nm for T-SOD, 412 nm for GSH-PX, and 532 nm for MDA at the end of the reaction.

All of the kits were from the Nanjing Jiancheng Bioengineering Institute, Nanjing, China.

### Histopathology

The liver and pancreas were excised from the mice and weighed. The tissue weight to body weight (BW) ratio was calculated. The sections were taken from each lobe of the pancreas and fixed with 10.0% formalin. Tissue dehydration was performed with acetone. After being embedded in paraffin, slices of 3-µm thickness were cut, and pancreas cells were examined in the HE-stained slides under an optical microscope.

### Immunohistochemical Assay

For the immunohistological staining of insulin, the pancreas samples were fixed in 4% paraformaldehyde. After that, pancreatic tissues were blocked in paraffin and then cut to 5-µm thickness. The primary antibody, anti-insulin (Wuhan Boster Bio-engineering Limited Company, Wuhan, China; 1:200) was incubated with tissues and then probed with a secondary antibody. After rinsing, the Elivison two-step method was performed for the immunohistochemical staining. An optical microscope was used to collect pictures.

### RT-PCR

The pancreas was stored in liquid nitrogen and was collected so that total RNA could be extracted according to the TRIzol RNA extraction method (TAKARA Biotechnology (Dalian) Co., Ltd.). RNA concentration was determined using a nanovolume spectrophotometer. The ratio of OD_260_/OD_280_ was 1.9–2.1, which suggested that the RNA was not contaminated. The amount of total RNA (ng/µL) was recorded.

The reverse transcription reaction system was assembled on ice according to the kit. There are 250–300 ng mRNA for reverse transcription reactions. The reaction system was set to 37°C for 15 min. The reverse transcriptase was then inactivated by heating to 85°C for 5 sec, and the cDNA was obtained. There are 2 µL cDNA for PCR reaction.

The primers were designed based on the gene sequences of PI3K (F: AAGCCATTGAGAAGAAAGGACTG, R: ATTTGGTAAGTCGGCGAGATAG), PDK-1 (F: CTGGGCGAGGAGGATCTG, R: CACAGCACGGGACGTTTC), AKT (F: TGTCTGCCCTGGACTACTTGC, R: GGCGTTCCGCAGAATGTC), FoxO1 (F: AGTGGATGGTGAAGAGCGTG, R: CTTTCCAGTTCCTTCATTCTGC), and β-actin (F: GAGACCTTCAACACCCCAGC, R: ATGTCACGCACGATTTCCC), and they were synthesized by Sangon Biotech (Shanghai) Co., Ltd., China.

PCR reaction conditions were as follows: 94°C for 4 min, 94°C for 20 sec, 60°C for 30 sec, and 72°C for 30 sec. Thirty-five cycles were conducted from the second step. Relative levels of the target mRNA expression were represented by the absorbance ratio of the target band to β-actin.

### Statistical analysis

The experimental data were expressed as the mean±SD and were analyzed with Origin 7.5 software. The one-way ANOVA was used to evaluate the difference between multiple groups, with *p*<0.05 considered as significant and *p*<0.01 as very significant.

## Results

### Effect of MBBP on weight

**Fig. 1:**
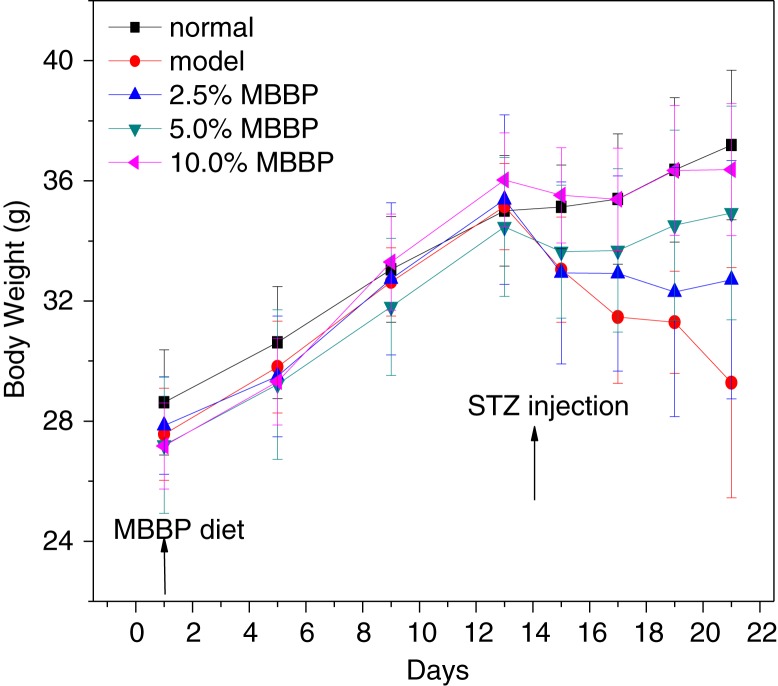
The effect of MBBP on the body weight of mice. Normal mice (no STZ injection) and model groups with STZ injection were fed with standard diet. Three MBBP-treated mice with STZ injection were administrated with standard diets containing 2.5, 5.0, and 10.0% MBBP, respectively. Data are mean ±SE values of 10 mice.

Abnormal regulation of insulin secretion has a significant effect on the weight of diabetic mice. During the treatment, the mice were all at growth state. As shown in Fig. 1, before injecting STZ, no obvious differences were found in the body weights of the five groups over 2 weeks. The weight of the normal group mice rose steadily and linearly during the 2 weeks, and the rate of increase somewhat slowed during the following week. We found that the weight of the model group was decreasing linearly. The weights of the 2.5 and 5.0% MBBP-treated mice were also decreasing somewhat, which indicated that growth and development were affected in these mice. After STZ injection, there were significant differences between the three MBBP groups. The weight growth trend of the MBBP group receiving the highest dose was similar to that of the normal group, and daily behaviors, such as feeding, drinking, and other activities, were almost the same as those in the normal group. Interestingly, MBBP administration significantly inhibited the weight loss of mice in a dose-dependent manner. Hence, these data indicate that MBBP had a beneficial preventive effect in inhibiting the weight loss of the STZ-induced mice.

### Effect of MBBP on the FBG

**Fig. 2:**
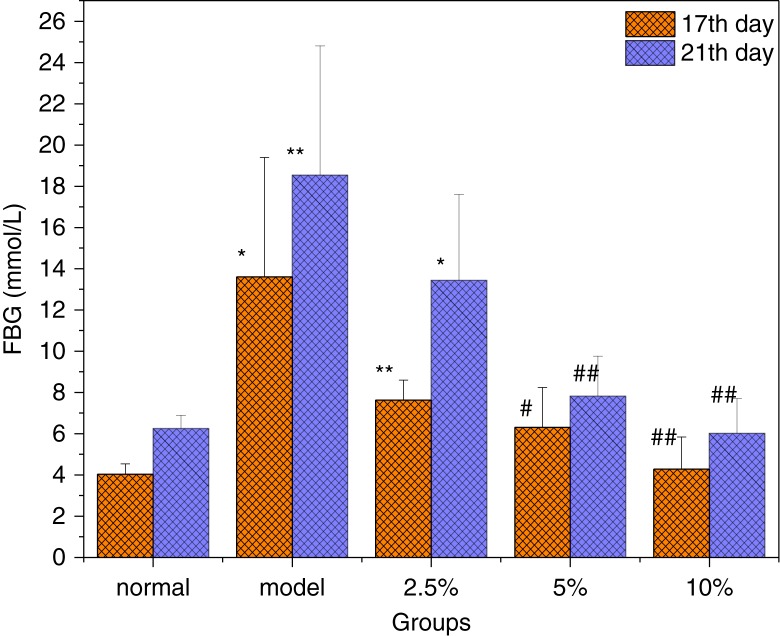
The effect of MBBP on FBG after STZ injection. Data are mean ±SE values (*n*=10). * and ** compared with the normal group with *p*<0.05 and *p*<0.01, respectively, significant differences. ^#^ and ^##^ compared with the model group with *p*<0.05 and *p*<0.01, respectively, significant differences.

STZ injection resulted in experimental diabetes, which is probably due to the impairment of β-cells in the islets of Langerhans (21). As shown in Fig. 2, after the mice fed with the MBBP diet for 2 weeks were injected with STZ on the 13th day, a significant increase in blood glucose in the model mice was observed compared with the normal group on the 17th and 21st days. These average glucose levels all reached 13.6 and 18.5 mmol/L, respectively. The FBG levels of MBBP-fed mice showed an obvious decrease compared with the model group, which is a positive correlation in a dose-dependent manner. Specifically, the FBGs of the 5.0 and 10.0% MBBP-treated groups were significantly lower than the model group (*p*<0.05 or *p*<0.01), and they were close to the normal control group. At the end (21st day) of the experiment, we calculated the incidence rates of diabetic mice using a standard value of FBG≥11.1 mmol/L to mean that induction of diabetes was successful. The results showed that the incidence rates of T2D mice induced by STZ decreased significantly by the oral administration of MBBP in a dose-dependent manner (Table 1). The model group had a higher incidence rate of diabetes, approximately 75%, while those of the 2.5 and 5.0% MBBP groups decreased to 66.7 and 12.5%, respectively. When 10.0% MBBP was administered, almost all of the mice were not diabetic. These results suggested that oral MBBP for 2 weeks prior to STZ injection could effectively prevent the incidence of STZ-induced T2D.

**Table 1:** The effect of MBBP on the incidence rate of diabetic mice (on the 21st day)

Group	Normal	Model	2.5% MBBP	5.0% MBBP	10.0% MBBP
Incidence rate of diabetes*	0	75.0%	66.7%	12.5%	0

* Diabetic mice means FBG≥11.1 mmol/L. All data were obtained from groups of 10 mice.

### Effect of MBBP on Serum Insulin

**Fig. 3:**
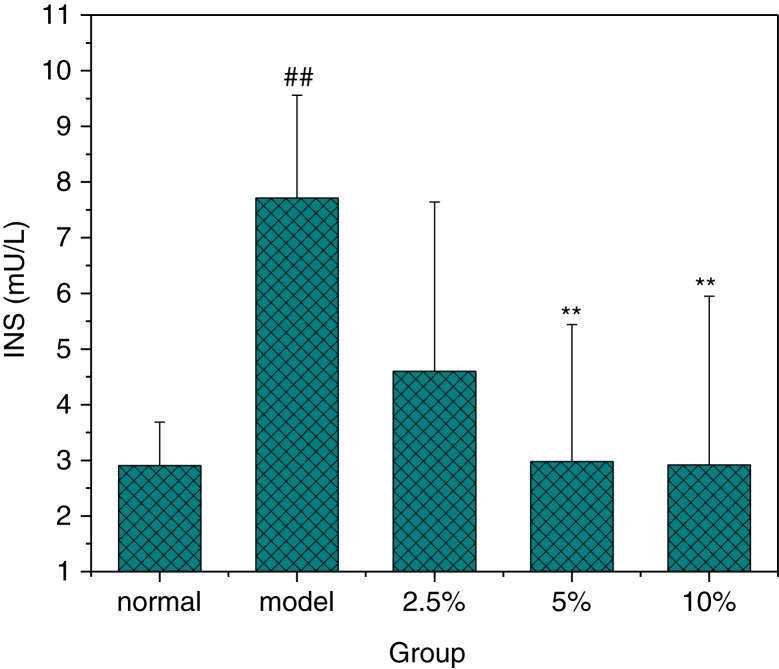
The effect of MBBP on insulin level after STZ injection. Data are mean ±SE values. ^##^ Compared with the normal group with *p*<0.01, significant differences. ** Compared with the model group with *p*<0.01, significant differences. All data were obtained from groups of at least three mice.

Insulin is important to regulate the FBG. Insulin resistance occurred when tissues were impaired and could not respond to insulin efficiently. As shown in Fig. 3, the insulin levels in model group were significantly higher than that in normal mice (*p*<0.01). Compared with the model group, the obvious reduction of insulin was observed in the MBBP-treatment groups. Specifically, the insulin of the 5.0 and 10.0% MBBP-treated groups were signally lower than the model group (*p*<0.01), and they were close to the normal mice. These data showed that MBBP could inhibit the abnormal increase of insulin and impede the occurrence of insulin resistance.

### Effect of MBBP on the coefficients of organs to body weight

Table 2 shows the coefficients of the liver and pancreas to body weight, which is expressed as milligrams (wet weight of tissues)/grams (fasted body weight). The coefficients of the pancreas and liver in the model group were significantly higher than those in the normal group (*p*<0.05 or *p*<0.01), indicating that the pancreas and liver swelled in those mice. Compared with the model group, the obvious reduction of these coefficients was observed in the pancreas and liver in the MBBP-treatment groups. Table 2 also shows that these coefficients of the pancreas and liver with the increased MBBP dose were gradually rescued in comparison with the model group, indicating that the pancreas and liver swelling were somewhat controlled. It was most significant in the 10.0% MBBP-treated group (*p*<0.01), and the coefficients of the pancreas and liver were close to the normal group, suggesting that the pancreas and liver were hardly affected by STZ. However, significant differences were not reached in the pancreas/BW of the 2.5% MBBP-fed group and the liver/BW of the 2.5 and 5.0% MBBP-fed groups. These data indicated that oral administration of MBBP could protect the pancreas and liver from enlargement induced by STZ and were confirmed by the following histopathological observations and immunohistochemical analyses of the pancreas of the mice.

**Table 2:** The effect of MBBP on the coefficients of organs to body weight, serum lipid levels, and antioxidant capacity

Group		Normal control	Model control	2.5% MBBP	5.0% MBBP	10.0% MBBP
Indexes	Pancreas/BW (mg/g)	5.97±0.78	7.38±0.53*	6.73±0.22	6.19±0.42*	5.37±0.33**
	Liver/BW (mg/g)	44.6±2.98	51.1±1.42**	48.3±4.70	49.35±5.96	43.8±2.98**
Serum lipid levels	TG (mmol/L)	2.15±0.33	2.98±0.12*	2.35±1.52	2.23±0.22*	2.04±0.14**
	HDL-C (mmol/L)	2.41±0.16	1.67±0.11*	1.89±0.63	2.28±0.28	2.52±0.20*
	CHOL (mmol/L)	2.86±0.49	2.66±0.30	2.92±0.98	3.33±0.37	3.49±0.04
	LDL-C (mmol/L)	0.43±0.13	0.28±0.02	0.45±0.08*	0.53±0.04**	0.66±0.01**
Antioxidant capacity	SOD (mmol/L)	74.84±5.26	63.71±5.233**	66.21±4.33**	70.89±1.56****	75.62±7.00**
	GSH-PX (mmol/L)	930.0±45.4	765.7±64.50**	800.9±34**	858.4±17.4****	903.0±10.00**
	MDA (mmol/L)	0.778±0.14	1.064±0.16**	0.898±0.02*	0.818±0.14*	0.781±0.08**

All data were obtained from groups of at least three mice.

* compared with the model group with *p*<0.05 and *p*<0.01, respectively, significant differences.

** compared with the model group with *p*<0.05 and *p*<0.01, respectively, significant differences.

** Compared with the normal group with *p*<0.01, significant difference.

### Effect of MBBP on serum lipid levels

To investigate the preventative effects of MBBP on lipid metabolism, the levels of TG, CHOL, HDL-C, and LDL-C were determined in the serum (Table 2). The TG levels of MBBP-fed mice improved significantly. The TG level of the 10.0% MBBP-fed group was effectively rescued and almost reached that of the normal group, with no significant difference (*p*>0.05). After STZ injection, the HDL-C levels in the model group were evidently lower than the normal group (*p*<0.05), while the HDL-C levels of the MBBP-fed groups rose in a dose-dependent manner. The level of the 10.0% MBBP-fed group continued to rise even more than the level of the normal group (*p*>0.05). These results showed that MBBP administration could resist the influence of STZ. Specifically, the TG and HDL-C in the 5.0 and 10.0% MBBP-fed groups were markedly maintained at a normal level, while the levels of the model group were abnormal (*p*<0.05 or *p*<0.01) in comparison with the normal controls. These results were similar to the results reported by Sendrayaperumal (22). Though the CHOL levels of both the MBBP-fed groups and the model group suffered a little fluctuation from STZ induction, these changes were not significant (*p*>0.05) in comparison with the normal group. After STZ injection, the LDL-C levels in the model group were significantly lower than the normal group. However, oral administration with different doses of MBBP did not make the LDL-C level decrease and increased it significantly in a dose-dependent manner, which showed a reverse trend to that in another report (23). The reason for this discrepancy should be investigated further.

### Effect of MBBP on antioxidant capacity

To explore the antioxidant capacity of mice fed with the MBBP diet, the levels of SOD, GSH-PX, and MDA were determined. As shown in Table 2, the SOD and GSH-PX activity in the model group declined markedly without MBBP treatment (*p*<0.05), indicating that STZ can decrease the antioxidant capacity of mice. With MBBP administration, the SOD and GSH-PX activity had higher levels than that of the model group, especially in the 5.0 and 10.0% MBBP-fed groups. The SOD and GSH-PX activities were hardly influenced by STZ, while the two enzyme activities of the model group changed significantly after the STZ injection (*p*<0.01). The MDA activity in the model group was markedly higher than that of the normal group (*p*<0.01), suggesting that superoxide anion-free radicals were increased after STZ injection. With increasing MBBP dosages, MDA levels decreased significantly compared with the model group (*p*<0.05 or *p*<0.01). The SOD, GSH-PX, and MDA activities of the 10.0% MBBP-treated group were very similar to the normal group, which meant that MBBP could eliminate the oxidative stress in diabetic mice induced by STZ. In other words, oral MBBP could enhance the antioxidation capabilities of mice and prevent oxidative damage in the liver induced by STZ.

### Histopathological observation

**Fig. 4:**
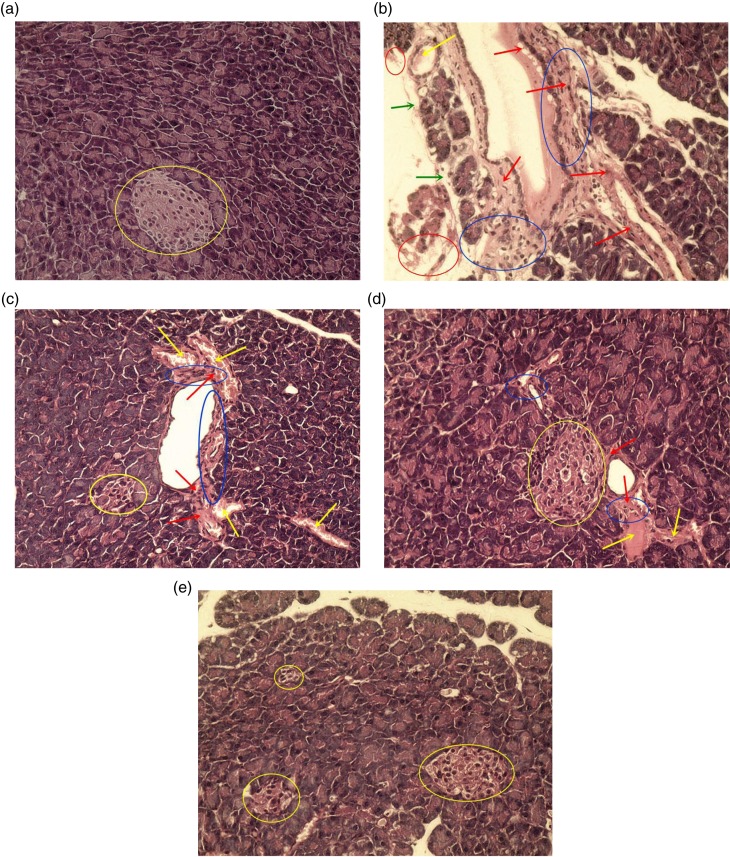
The effect of MBBP on pathologic tissue in the pancreas (×400). The graphs were representative of the 10 mice in each group. (a) normal mice fed with a standard diet; (b) model mice fed with a standard diet; (c–e): treated mice fed with 2.5, 5.0, and 10.0% MBBP diets, respectively; blue circle means minute inflammatory cell infiltration; yellow circle indicates pancreas islet; red arrow means slight interstitial proliferation and angionecrosis; blue arrow means degeneration of superficial adipocytes; yellow arrows mean tiny congestion of vascellum and prominent vasodilation.

The pancreas is an important organ for regulating blood glucose and secreting insulin. To elucidate the preventative effects of MBBP-treatment on the STZ-induced diabetic mice, the pancreatic islets were examined histologically. The pancreatic tissues were subjected to HE staining. The normal pancreatic samples had an intact architecture and regularly arranged pancreatic cells, and the cell nuclei were clearly visible (Fig. 4a). In the model group, standard diet-fed mice exhibited severe pathological changes, including infiltration of inflammatory cells, serious hemorrhagic necrosis of partly pancreatic cells and peripheral adipose tissue, severe interstitial proliferation and angionecrosis, degeneration of superficial adipocytes, and congestion of vascellum and prominent vasodilation. The pancreas showed partly degenerative and necrotic changes. The islets atrophied to the point where pancreas islets could not be observed under the microscope (Fig. 4b). With 2.5% MBBP-fed mice for 2 weeks, Fig. 4c shows a slight inflammatory cell infiltration, light interstitial proliferation and angionecrosis, and congestion of the vascellum and prominent vasodilation. Small pancreatic islets were found. As shown in Fig. 4d, mice pancreas had minute inflammatory cell infiltration, tiny congestion in the vascellum and prominent vasodilation, as well as slight interstitial proliferation and angionecrosis. The intact islet retained a large volume in the 5.0% MBBP-treated group. In the 10.0% MBBP-fed group, the mice had hardly any pathological changes. The pancreatic cells lined up tightly, the same as normal mice (Fig. 4e). The results suggested that MBBP could protect the pancreas and islets from the STZ-induced damage.

### Insulin protein expression in pancreas

**Fig. 5:**
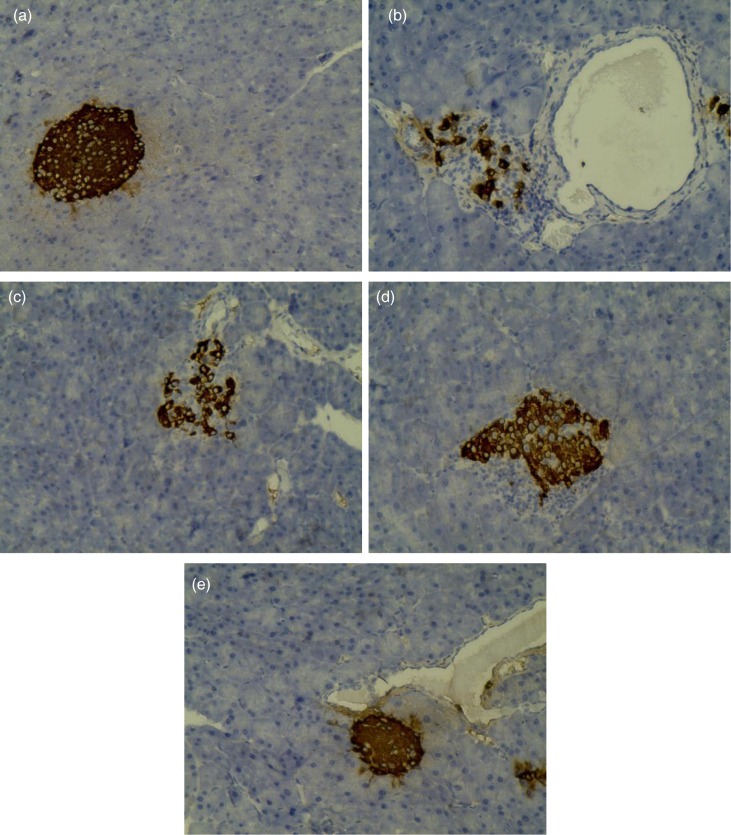
Insulin immunohistochemistry of pancreas in mice (×400). The graphs were representative of the 10 mice in each group. (a) normal group; (b) model group; (c) 2.5% MBBP-fed group; (d) 5.0% MBBP-fed group; (e) 10.0% MBBP-fed group.

To demonstrate the results of the above experiments, that MBBP administration could decrease the incidence of STZ-induced T2D mice and affect the metabolism of glucose and lipids, an immunohistochemical method was used to study the protection of MBBP to the pancreas and islet cells. Figure 5 demonstrates the immunohistochemical effects of MBBP on the pancreases of the experimental mice. The insulin protein in normal islet cells was dyed into dark brown granulation in Fig. 5. In the normal group (Fig. 5a), the islets show a normal structure with insulin-secreting β-cells. After STZ injection, the model group mice fed with the same standard diet exhibited markedly impaired insulin-secreting β-cells in comparison with the control group. The dark brown granulation showed abnormal alterations (Fig. 5b). The expression of insulin protein in MBBP-fed mice was significantly better than that of the model group. Moreover, the expression of insulin was positively correlated with MBBP dose (Fig. 5c–e). Specifically, intact islets were observed in the pancreas of 10.0% MBBP-fed mice (Fig. 5e). These results show that a high dose of MBBP oral administration can inhibit oxidative impairment caused by STZ to the pancreatic and islet cells in mice.

### Effect of MBBP on the PI3K/Akt signaling pathway in the pancreas

The PI3K/Akt pathway plays a crucial role in the signal transduction of insulin. PI3K/Akt activation by insulin might contribute to the development of insulin resistance (24). Impaired insulin-stimulated glucose uptake might occur due to the lack of a coordinated decrease in insulin-mediated insulin receptor substrate 1 (IRS1) phosphorylation or PI3K activation (25). PI3K activates PDK1 through phosphorylation, and p-PDK1 can activate Akt phosphorylation. Active Akt makes FoxO1 phosphorylative and, in turn, represses the transcriptional activation of FoxO1. That results in the redistribution of FoxO1 from the cell nucleus to the cell plasma, eventually exerting a protective effect on β-cells (26, 27). FoxO1 is a crucial nuclear transcription factor that inhibits or activates target gene transcription after insulin-induced phosphorylation.

**Fig. 6:**
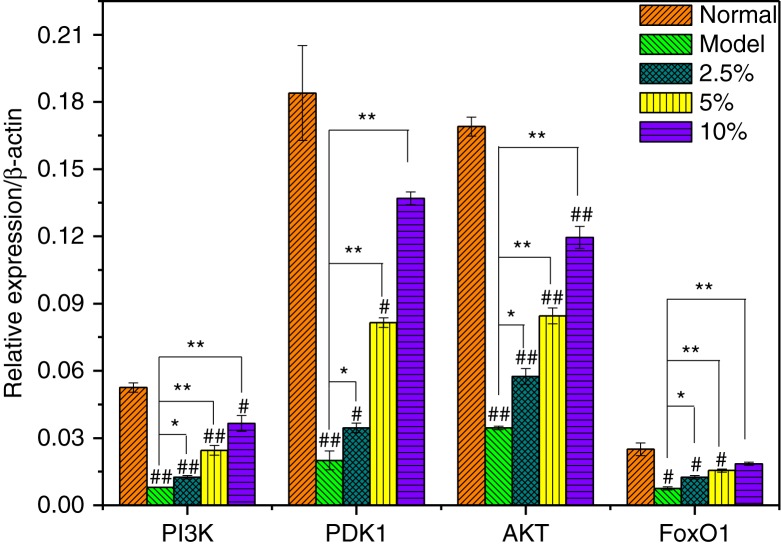
The preventive effect of MBBP to PI3K, PDK-1, AKT, and FoxO1 mRNA expression in pancreas. ^#^ and ^##^ compared with normal group with *p*<0.05 and *p*<0.01, respectively, significant differences; * and ** compared with model group with *p*<0.05 and *p*<0.01, respectively, significant differences. All data were obtained from groups of at least two mice.

To explore the protection of MBBP against diabetes involved in the PI3K/Akt signaling pathway, the levels of the insulin-related genes, PI3K, PDK-1, AKT, and FoxO1, were evaluated (Fig. 6). Markedly diminished expressions of PI3K were observed in model group mice compared with the normal group (*p*<0.05). In the three MBBP-treated groups, the expression levels of PI3K gradually recovered in a dose-dependent manner, but their levels were all lower than that of normal mice (*p*<0.05 or *p*<0.01). Of course, PI3K expression in the 10.0% MBBP-treated group was still slightly lower than that of normal mice but much higher than that of model mice (*p*<0.01).

Compared with normal mice, a significant down-regulation of PDK1 mRNA expression was observed in the pancreas in STZ-injected mice (*p*<0.01). When given an oral administration of MBBP, the expression of the PDK1 gene significantly improved. The expression levels increased linearly with MBBP in a dose-dependent manner compared with that of the model group (*p*<0.05 or *p*<0.01), indicating that MBBP administration could protect PDK1 expression against STZ. The expression of the AKT gene in the model and MBBP-treated mice was almost similar to that of the PDK1 gene. The expressions of the two genes in the 10.0% MBBP-fed mice were much higher than those of the model mice and almost arrived at the level of normal mice.

In addition, administration with MBBP also preserved the expression of FoxO1 mRNA and avoided the effects induced by STZ in a dose-dependent manner (*p*<0.05 or *p*<0.01). A significantly down-regulated FoxO1 mRNA expression could be observed in the pancreas in STZ-injected mice (*p*<0.01). In the 10.0% MBBP-treated group, the expression level of FoxO1 mRNA was very close to normal group mice (*p*>0.05).

In general, the expressions of the above four genes in the pancreatic cells of model mice suffered significantly from STZ, while oral administration of MBBP-fed mice, especially the highest dose, could almost prevent the serious damage to pancreatic cells from STZ, and their expression levels were almost returned to normal.

## Discussion

The prevalence of diabetes has been increasing rapidly over the past decade and is now considered a global epidemic and a metabolic disorder. Overall, 92.4 million adults have diabetes according to a report in 2010 (28). In addition, 148.2 million adults have pre-diabetes, which is an important risk factor for the development of cardiovascular disease and overt diabetes (29, 30).

In recent years, with the rise of green drugs, many studies had been devoted to searching for safe and effective drugs from natural materials for diabetes prevention. The search for natural compounds, preferably in the diet, has received wide attention as a preventive approach to delay the onset of diabetes and the appearance of diabetic complications. Several studies have been performed to evaluate the hypoglycemic effect and antioxidation of mulberry branch bark extract (31, 32). The hypoglycemic, hypolipidemic, and antioxidative effects of flavonoids (33), stilbenes (34), polysaccharides (35), alkaloids (30, 36), and other components in mulberry branch bark have been approved. However, very little research has focused on the diabetes prevention of the mulberry leaf, branch, root, or fruit. Recently, Liu et al. published a study of glucosidase inhibitors and explored the effect of mulberry branch bark extract on STZ-diabetic mice (13). In this paper, the ethanol and water extract from the mulberry branch bark effectively lowered the blood glucose of STZ-induced T2D mice.

Diabetes is a chronic disease that occurs either when the pancreas does not produce enough insulin or when the body cannot effectively use the insulin produced by the pancreas. STZ can result in the damage of the insulin-producing beta cells in the mammalian pancreas, and it has also been used for inducing diabetes in experimental animals for a long time (37, 38). STZ not only causes glucose metabolism disorders but it also induces insulin resistance, which results in an increase of the blood insulin level (39).

To date, many studies have shown that abnormal regulation of insulin secretion has a significant effect on the weight loss of diabetic patients (40). With MBBP administration for 2 weeks, the weight in MBBP-fed mice was as normal as that in the normal control group. However, after STZ injection, the weight loss of the MBBP-treated group was significantly lower than that in the model group in a dose-dependent manner. The same situation was observed with FBG and insulin levels; the FBGs and insulin of the MBBP-fed group were significantly lower than those of the model group. This experimental result shows that MBBP can inhibit weight loss and prevent the FBG from rising after STZ injection. It's important that MBBP can reduce insulin resistance, which results in a decrease of the serum insulin level. Above all, the incidence rates of diabetes induced by STZ were reduced by pre-treatment with MBBP in a dose-dependent manner.

Hypertriglyceridemia and hypercholesterolemia are two major problems in diabetic patients, and they are responsible for atherosclerosis, coronary heart disease, and the secondary complications of diabetes (41). Several reports show that an increased HDL-C content is associated with reduced coronary risk (42, 43). In this paper, compared with the model group, MBBP-treatment significantly maintained the levels of TG and HDL-C in response to STZ damage. However, the levels of CHOL and LDL-C were abnormal in the MBBP-treatment group, which is contrary to other reports (20, 44). These data show that MBBP-treatment has a benefit for TG and HDL-C but not for CHOL and LDL-C. The reasons for these conflicting results are not clear and should be approached in the future.

STZ, an analogue of GlcNAc, acts as a NO donor, and many investigators postulate that NO is involved in its biological function (45, 46). Because of the chemical properties of NO, it can interact with superoxide anion to form ONOO^−^, whose oxidizability is 200 times that of H_2_O_2_ (47). Reactive oxygen species (ROS) and oxidative stress have been shown to be one of the most important regulatory mechanisms of pathological events, including diabetes (48). SOD and GSH-PX are primary antioxidant enzymes of detoxification in cells, and they can effectively relieve peroxide damage and regulate the balance between free radical production and antioxidant defense (49). Moreover, MDA is the lipid end product generated by peroxidation, and MDA can gradually inflict damage to the cellular membrane structures, resulting in cellular dysfunction and cytotoxicity (50). These experiment results showed that STZ injection could significantly reduce the activities of SOD and GSH-PX (*p*<0.01) and increase MDA production in the livers of mice (*p*<0.01). Importantly, MBBP could increase the contents of SOD and GSH-PX, and reduce the MDA content. These data illustrate fully that MBBP protected against these abnormalities in the antioxidant systems in mice.

MBBP improved the antioxidant capacity in mice. Hence, as shown in Fig. 4, β-cells in MBBP-fed mice did not exhibit STZ-induced histological toxicity, and it is very probable that the MBBP-enhanced antioxidant capacity protected the pancreatic β-cells from STZ toxicity. Diabetes arises from irreversible damage of the β-cells of the islets in the pancreas, causing a reduction or deregulation of insulin (51). As shown in Fig. 5, the insulin-secreting β-cells were preserved significantly in the pancreas of the MBBP-fed mice. Therefore, the increasing hyperglycemic and hypolipidemic activities in mice were mainly contributed by active substances in the MBBP, which could protect against STZ-induced destruction of the functional β-cells and stimulate insulin secretion.

In the present study, the pancreatic PI3K/Akt/FoxO1 signaling pathway exhibited abnormal changes in model mice induced by STZ, as manifested by PI3K, PDK1, Akt, and FoxO1 expression. As shown in Fig. 6, oral administration of MBBP protected the genes of the PI3K/Akt/FoxO1 signaling pathway to rescue gluconeogenesis and glycogen synthesis, so that a normal level of blood glucose was maintained.
